# Supplementary material for: Microneutralization assay titer correlates analysis in two phase 3 trials of the CYD-TDV tetravalent dengue vaccine in Asia and Latin America
Source: PLoS One. 2020 Jun 15;15(6):e0234236. doi: 10.1371/journal.pone.0234236 (PMC7295445; doi:10.1371/journal.pone.0234236)
Supplement: S1 Table — (DOCX) [file pone.0234236.s001.docx]

S1 Table. Correlations between MN and PRNT_50_ titers

| A. Baseline MN and baseline PRNT_50_ titers | | | | |
| --- | --- | --- | --- | --- |
| Treatment Groups | Readout | *N* | Correlation | 95% CI |
| Placebo & Vaccine | Average^1^ | 493 | 0.96 | (0.95, 0.97) |
| Placebo & Vaccine | DENV-1 | 491 | 0.94 | (0.93, 0.96) |
| Placebo & Vaccine | DENV-2 | 492 | 0.90 | (0.88, 0.92) |
| Placebo & Vaccine | DENV-3 | 491 | 0.94 | (0.92, 0.96) |
| Placebo & Vaccine | DENV-4 | 492 | 0.84 | (0.81, 0.88) |
| B. Month 13 MN and Month 13 PRNT_50_ titers | | | | |
| Treatment | Readout | *N* | Correlation | 95% CI |
| Placebo | Average^1^ | 570 | 0.95 | (0.93, 0.96) |
| Placebo | DENV-1 | 570 | 0.94 | (0.92, 0.96) |
| Placebo | DENV-2 | 569 | 0.91 | (0.90, 0.93) |
| Placebo | DENV-3 | 569 | 0.93 | (0.90, 0.95) |
| Placebo | DENV-4 | 570 | 0.87 | (0.84, 0.90) |
| Vaccine | Average^1^ | 1207 | 0.94 | (0.93, 0.95) |
| Vaccine | DENV-1 | 1207 | 0.93 | (0.91, 0.94) |
| Vaccine | DENV-2 | 1207 | 0.88 | (0.86, 0.90) |
| Vaccine | DENV-3 | 1206 | 0.91 | (0.89, 0.92) |
| Vaccine | DENV-4 | 1205 | 0.81 | (0.78, 0.83) |

^1^Average titer is the average log_10_ titer across all 4 serotypes.

Rank correlation coefficients between baseline or Month 13 titers measured by the microneutralization (MN) assay and baseline or Month 13 titers measured by the PRNT_50_ assay were calculated as described by Liu et al. (Q. Liu et al., Biometrics 2017, doi:10.1111/biom.128122017) using the R package PResiduals. Calculations were performed by adjusting for age (as a continuous variable) and country. For (A), the analysis was performed for pooled vaccine and placebo recipients in the immunogenicity subset cases and controls among CYD14 & CYD15 9–16-year-olds. For (B), the analysis was performed separately for vaccine and placebo recipients in the immunogenicity subset controls among CYD14 & CYD15 9–16-year-olds. *N* indicates the number of participants for whom both (A) baseline MN and baseline PRNT_50_ titers or (B) Month 13 MN and Month 13 PRNT_50_ titers were available.
