# Supplementary material for: Microneutralization assay titer correlates analysis in two phase 3 trials of the CYD-TDV tetravalent dengue vaccine in Asia and Latin America
Source: PLoS One. 2020 Jun 15;15(6):e0234236. doi: 10.1371/journal.pone.0234236 (PMC7295445; doi:10.1371/journal.pone.0234236)
Supplement: S2 Table — (DOCX) [file pone.0234236.s002.docx]

S2 Table. Concordance with respect to baseline serostatus classification in the CYD14 and CYD15 trials for the MN and PRNT_50_ assays.

| 1. CYD14 and CYD15 combined (2–16-year-olds)^1^ | | | | |
| --- | --- | --- | --- | --- |
|  | MN- | MN+ | PRNT_50_ Summary |  |
| PRNT_50_- | 429 | 2 | 431 (24%) | Specificity _MN_: 99.5%* |
| PRNT_50_+ | 134 | 1203 | 1337 (76%) | Sensitivity _MN_: 90.0% |
| MN Summary | 563 (32%) | 1205 (68%) | 92.3% (1632/1768) | κ = 0.81 |
| 1. CYD14 (2–14-year-olds)^1^ | | | | |
|  | MN- | MN+ | PRNT_50_ Summary |  |
| PRNT_50_- | 248 | 0 | 248 (32% SN) | Specificity _MN_= 100.0% |
| PRNT_50_+ | 74 | 444 | 518 (68% SP) | Sensitivity _MN_= 85.7% |
| MN Summary | 322 (42%) | 444 (58%) | 90.3% (692/766) | κ = 0.80 |
| 1. CYD15 (9–16-year-olds)^1^ | | | | |
|  | MN- | MN+ | PRNT_50_ Summary |  |
| PRNT_50_- | 181 | 2 | 183 (18%) | Specificity _MN_= 98.9% |
| PRNT_50_+ | 60 | 759 | 819 (82%) | Sensitivity _MN_= 92.7% |
| MN Summary | 241 (24%) | 761 (76%) | 93.8% (940/1002) | κ = 0.82 |
| 1. CYD14 and CYD15 combined (≥ 9 years old)^2^ | | | | |
|  | MN- | MN+ | PRNT_50_ Summary |  |
| PRNT_50_- | 286 | 2 | 288 (21%) | Specificity _MN_= 99.3% (286/288) |
| PRNT_50_+ | 97 | 968 | 1065 (79%) | Sensitivity _MN_= 90.9% (97/1065) |
| MN Summary | 383 (28%) | 970 (72%) | 93% (1254/1353) | κ = 0.81 |
| 1. CYD14, 6–8-year-olds^2^ | | | | |
|  | MN- | MN+ | PRNT_50_ Summary |  |
| PRNT_50_- | 54 | 0 | 54 (35%) | Specificity _MN_ = 100% (54/54) |
| PRNT_50_+ | 14 | 88 | 102 (65%) | Sensitivity _MN_= 86.3% (14/102) |
| MN Summary | 68 (44%) | 88 (56%) | 91% (142/156) | κ = 0.81 |
| 1. CYD14, 2–5-year-olds^2^ | | | | |
|  | MN- | MN+ | PRNT_50_ Summary |  |
| PRNT_50_- | 89 | 0 | 89 (34%) | Specificity _MN_= 100% (89/89) |
| PRNT_50_+ | 23 | 148 | 171 (66%) | Sensitivity _MN_= 86.5% (23/171) |
| MN Summary | 112 (43%) | 148 (57%) | 91% (237/260) | κ = 0.82 |

^1^Analyses included Month 0 measurements for participants pooled across the vaccine and placebo groups and M13 measurements for participants in the placebo group.

^2^Analyses included Month 0 measurements for participants pooled across the vaccine and placebo groups.

*The PRNT_50_ assay was assumed as the gold standard for serostatus determination in calculations of MN specificity and sensitivity.

Seronegative = titer < 10 to all four serotypes. Seropositive = titer > 10 to at least one serotype.

κ = Cohen’s kappa.
