# Supplementary material for: Microneutralization assay titer correlates analysis in two phase 3 trials of the CYD-TDV tetravalent dengue vaccine in Asia and Latin America
Source: PLoS One. 2020 Jun 15;15(6):e0234236. doi: 10.1371/journal.pone.0234236 (PMC7295445; doi:10.1371/journal.pone.0234236)
Supplement: S3 Table — For the PRNT50 assay, the CoR analyses were performed on all CYD14 and CYD15 data, as reported in Moodie et al. [11], and for the MN assay, the CoR analyses were restricted to 9–16-year-olds from CYD14 and 15. (DOCX) [file pone.0234236.s003.docx]

S3 Table. Logistic regression estimated odds ratios (ORs) (95% CIs) of matched-serotype VCD per log_10_ increase in Month 13 neutralizing antibody titer as measured by each assay, pooling data from the CYD14 and CYD15 studies together. Modeling was performed as for the correlates of risk modeling, with adjustment for potential confounders including age, sex, and country. The proportions of the treatment effect captured by the immune response in (A) were determined as described in Kobayashi and Kuroki (F. Kobayashi and M. Kuroki. Stat Med 33:3338-53, 2014). A marker is supported to be a valid surrogate endpoint if (1) the titer OR in Model 1 and the Trt OR in Model 2 both differ from one; (2) the Trt x titer ratio of Model 4 equals one (no interaction); and (3) the titer OR in Model 3 differs from one and the Trt OR in Model 3 equals one (“full mediation” by titer).

| A. Modeling with immune response = Month 13 PRNT_50_ titer | | | | |
| --- | --- | --- | --- | --- |
| DENV-1 PRNT_50_ | | | | |
|  | Placebo Group Only | Placebo and Vaccine Groups | | |
|  | Model 1 | Model 2 | Model 3 | Model 4 |
| Intercept | 0.14 (0.04, 0.49) | 0.01 (0.00, 0.02) | 0.05 (0.02, 0.15) | 0.05 (0.02, 0.17) |
| DENV-1 | 0.26 (0.19, 0.35) | - | 0.30 (0.24, 0.36) | 0.27 (0.20, 0.37) |
| Trt | - | 0.49 (0.36, 0.67) | 1.01 (0.71, 1.42) | 0.81 (0.42, 1.57) |
| Trt x DENV-1* | - | - | - | 1.16 (0.80, 1.69) |
| Model 4 supports no effect modification of the DENV-1 titer correlate of risk by treatment group and Model 3 supports that treatment group does not predict DENV-1 VCD after controlling for DENV-1 titer; thus the data are consistent with the Prentice criteria and the Prentice definition may hold. | | | | |
| Predicted VE assuming a valid Prentice surrogate = 0.50 | | | | |
| Empirical VE ignoring immune response variable = 0.56 | | | | |
| Proportion of treatment effect captured by immune response = 1, 95% CI = (0.85, 1) | | | | |
| DENV-2 PRNT_50_ | | | | |
|  | Placebo Group Only | Placebo and Vaccine Groups | | |
|  | Model 1 | Model 2 | Model 3 | Model 4 |
| Intercept | 0.00 (0.00, 0.00) | 0.00 (0.00, 0.01) | 0.02 (0.00, 0.07) | 0.01 (0.00, 0.04) |
| DENV-2 | 0.70 (0.50, 0.98) | - | 0.42 (0.32, 0.55) | 0.71 (0.52, 0.96) |
| Trt | - | 0.68 (0.47, 0.99) | 1.41 (0.84, 2.38) | 11.83 (4.44, 31.50) |
| Trt x DENV-2* | - | - | - | 0.32 (0.20, 0.51) |
| Model 4 supports effect modification of the DENV-2 correlate of risk by treatment group, indicating that the Prentice definition does not hold. | | | | |
| Predicted VE assuming a valid Prentice surrogate =0.49 | | | | |
| Empirical VE ignoring immune response variable = 0.40 | | | | |
| Proportion of treatment effect captured by immune response = 0.89, 95% CI = (0.57, 1) | | | | |
| DENV-3 PRNT_50_ | | | | |
|  | Placebo Group Only | Placebo and Vaccine Groups | | |
|  | Model 1 | Model 2 | Model 3 | Model 4 |
| Intercept | 0.00 (0.00, 0.00) | 0.01 (0.00, 0.02) | 0.03 (0.01, 0.13) | 0.03 (0.01, 0.13) |
| DENV-3 | 0.43 (0.34, 0.55) | - | 0.43 (0.35, 0.53) | 0.43 (0.34, 0.54) |
| Trt | - | 0.28 (0.19, 0.41) | 0.49 (0.33, 0.74) | 0.51 (0.18, 1.45) |
| Trt x DENV-3* | - | - | - | 0.98 (0.64, 1.50) |
| Model 4 supports no effect modification of the DENV-3 correlate of risk by treatment group and Model 3 shows that treatment still predicts DENV-3 VCD accounting for DENV-3 titer, indicating that the Prentice definition does not hold. | | | | |
| Predicted VE assuming a valid Prentice surrogate =0.51 | | | | |
| Empirical VE ignoring immune response variable = 0.75 | | | | |
| Proportion of treatment effect captured by immune response = 0.67, 95% CI = (0.38, 0.93) | | | | |
| DENV-4 PRNT_50_ | | | | |
|  | Placebo Group Only | Placebo and Vaccine Groups | | |
|  | Model 1 | Model 2 | Model 3 | Model 4 |
| Intercept | 0.02 (0.00, 0.15) | 0.00 (0.00, 0.02) | 0.02 (0.00, 0.14) | 0.01 (0.00, 0.11) |
| DENV-4 | 0.36 (0.25, 0.52) | - | 0.30 (0.21, 0.43) | 0.38 (0.27, 0.55) |
| Trt | - | 0.19 (0.11, 0.32) | 0.51 (0.27, 0.97) | 1.72 (0.43, 6.87) |
| Trt x DENV-4* | - | - | - | 0.49 (0.24, 1.00) |
| Model 4 supports possible effect modification of the DENV-4 correlate of risk by treatment group, suggesting some departure from the Prentice definition. | | | | |
| Predicted VE assuming a valid Prentice surrogate = 0.71 | | | | |
| Empirical VE ignoring immune response variable = 0.81 | | | | |
| Proportion of treatment effect captured by immune response = 0.93, 95% CI = (0.71, 1) | | | | |
| B. Modeling with immune response = Month 13 MN titer | | | | |
| DENV-1 MN | | | | |
|  | Placebo Group Only | Placebo and Vaccine Groups | | |
|  | Model 1 | Model 2 | Model 3 | Model 4 |
| Intercept | 0.21 (0.05, 0.80) | 0.01 (0.00, 0.02) | 0.05 (0.02, 0.16) | 0.06 (0.02, 0.20) |
| DENV-1 | 0.14 (0.08, 0.24) | - | 0.19 (0.14, 0.25) | 0.15 (0.09, 0.25) |
| Trt | - | 0.51 (0.37, 0.71) | 1.01 (0.71, 1.43) | 0.69 (0.33, 1.45) |
| Trt x DENV-1* | - | - | - | 1.44 (0.77, 2.69) |
| Model 4 is consistent with no effect modification of the DENV-1 correlate of risk by treatment group and Model 3 supports that treatment group does not predict DENV-1 VCD after controlling for DENV-1 titer; thus the data are consistent with the Prentice criteria and the Prentice definition may hold | | | | |
| Predicted VE assuming a valid Prentice surrogate = 0.48 | | | | |
| Empirical VE ignoring immune response variable = 0.56 | | | | |
| DENV-2 MN | | | | |
|  | Placebo Group Only | Placebo and Vaccine Groups | | |
|  | Model 1 | Model 2 | Model 3 | Model 4 |
| Intercept | 0.00 (0.00, 0.00) | 0.00 (0.00, 0.01) | 0.02 (0.00, 0.08) | 0.01 (0.00, 0.05) |
| DENV-2 | 0.42 (0.22, 0.81) | - | 0.22 (0.15, 0.35) | 0.42 (0.22, 0.79) |
| Trt | - | 0.71 (0.48, 1.03) | 1.23 (0.80, 1.90) | 3.38 (1.32, 8.63) |
| Trt x DENV-2* | - | - | - | 0.38 (0.17, 0.84) |
| Model 4 supports effect modification of the DENV-2 correlate of risk by treatment group, indicating that the Prentice definition does not hold. | | | | |
| Predicted VE assuming a valid Prentice surrogate = 0.42 | | | | |
| Empirical VE ignoring immune response variable = 0.40 | | | | |
| DENV-3 MN | | | | |
|  | Placebo Group Only | Placebo and Vaccine Groups | | |
|  | Model 1 | Model 2 | Model 3 | Model 4 |
| Intercept | 0.00 (0.00, 0.00) | 0.01 (0.00, 0.03) | 0.04 (0.01, 0.19) | 0.04 (0.01, 0.19) |
| DENV-3 | 0.30 (0.22, 0.40) | - | 0.29 (0.22, 0.38) | 0.29 (0.22, 0.40) |
| Trt | - | 0.29 (0.20, 0.43) | 0.61 (0.40, 0.92) | 0.64 (0.23, 1.82) |
| Trt x DENV-3* | - | - | - | 0.97 (0.57, 1.63) |
| Model 4 supports no effect modification of the DENV-3 correlate of risk by treatment group and Model 3 supports that treatment predicts DENV-3 VCD after accounting for DENV-3, indicating that the Prentice definition does not hold. | | | | |
| Predicted VE assuming a valid Prentice surrogate = 0.57 | | | | |
| Empirical VE ignoring immune response variable = 0.74 | | | | |
| DENV-4 MN | | | | |
|  | Placebo Group Only | Placebo and Vaccine Groups | | |
|  | Model 1 | Model 2 | Model 3 | Model 4 |
| Intercept | 0.03 (0.00, 0.24) | 0.00 (0.00, 0.02) | 0.03 (0.00, 0.22) | 0.02 (0.00, 0.15) |
| DENV-4 | 0.28 (0.18, 0.44) | - | 0.20 (0.13, 0.31) | 0.29 (0.19, 0.46) |
| Trt | - | 0.19 (0.12, 0.33) | 0.65 (0.34, 1.25) | 2.53 (0.61, 10.52) |
| Trt x DENV-4* | - | - | - | 0.38 (0.16, 0.92) |
| Model 4 supports effect modification of the DENV-4 correlate of risk by treatment group, indicating that the Prentice definition does not hold. | | | | |
| Predicted VE assuming a valid Prentice surrogate = 0.77 | | | | |
| Empirical VE ignoring immune response variable = 0.79 | | | | |

Trt, treatment group (vaccine or placebo).

*A ratio (vaccine/placebo) of estimated odds ratios is shown.
