# Supplementary material for: Microneutralization assay titer correlates analysis in two phase 3 trials of the CYD-TDV tetravalent dengue vaccine in Asia and Latin America
Source: PLoS One. 2020 Jun 15;15(6):e0234236. doi: 10.1371/journal.pone.0234236 (PMC7295445; doi:10.1371/journal.pone.0234236)
Supplement: S4 Table — (DOCX) [file pone.0234236.s004.docx]

S4 Table. Distinct Input Variable Sets Used for Learning Algorithms and Learning Algorithms in the Super-Learner Library of Estimators of the Conditional Probability of DENV-Any.

| A. Input Variable Sets Used for Learning Algorithms | | | |  |  |
| --- | --- | --- | --- | --- | --- |
| Variable Set Name | | Variables | |  |  |
| 1. Demo | | Age (protocol-specified categories: 2-5, 6-8, 9-14 for CYD14; 9-11, 12-16 for CYD15), Sex, 4 serotype rate/prevalence variables (the fractions of DENV-Any cases in placebo recipients that are of serotype k, for k=1,2,3,4) | |  |  |
| 2. Demo + MN | | Demo + Month 13 MN readouts: 4 individual serotype titers, average, minimum, maximum | |  |  |
| 3. Demo + PRNT_50_ | | Demo + Month 13 PRNT_50_ readouts: 4 individual serotype titers, average, minimum, maximum | |  |  |
| 4. Demo + MN + PRNT_50_ | | Union of variables in Demo, MN and PRNT_50_ | |  |  |
| B. Learning Algorithms^a^ in the Super-Learner Library of Estimators of the Conditional Probability of DENV-Any | | | |  |  |
| Algorithm Type^b^ | | Input Variable Sets | | Algorithm Type Input Variable Sets Screens^c^ | |
| SL.mean | | None | | None (Base model: No covariates) | |
| SL.glm | | 1, 2, 3, 4 | | All, Lasso, univariate log. regression p-value < 0.10, Low-collinearity for 2, 3, 4 | |
| SL.glm.interaction | | 1, 2, 3, 4 | | All, Lasso, univariate log. regression p-value < 0.10, Low-collinearity for 2, 3, 4 | |
| SL.step (stepwise) | | 1, 2, 3, 4 | | All, Lasso, univariate log. regression p-value < 0.10, Low-collinearity for 2, 3, 4 | |
| SL.glmnet (lasso) | | 1, 2, 3, 4 | | All, Lasso, univariate log. regression p-value < 0.10, Low-collinearity for 2, 3, 4 | |
| SL.bayesglm | | 1, 2, 3, 4 | | All, Lasso, univariate log. regression p-value < 0.10, Low-collinearity for 2, 3, 4 | |
| SL.gam | | 1, 2, 3, 4 | | Lasso, univariate log. regression p-value < 0.10, Low-collinearity for 2, 3, 4 | |
| SL.nnet (neural net) | | 1, 2, 3, 4 | | Lasso, univariate log. regression p-value < 0.10, Low-collinearity for 2, 3, 4 | |
| SL.polymars | | 1, 2, 3, 4 | | Lasso, univariate log. regression p-value < 0.10, Low-collinearity for 2, 3, 4 | |

^a^ All algorithm type names are built in algorithms in the Super-Learner R package available at CRAN, and have documentation within the package. All algorithms properly adjusted for IPCW weights in model fitting and prediction.

^b^ SL.mean is a base reference model using no input variables; SL.glm is a logistic regression model fit to all input variables; SL.glm.interaction is a logistic regression model including all input variables together with all pairwise-interaction variables; SL.step is a logistic regression model with step-wise model selection with best model selected by the AIC criterion; SL.glmnet is the lasso that includes variables with non-zero estimated coefficients in the default implementation of SL.glmnet that optimizes the tuning parameter via cross-validation; SL.bayesglm is Bayesian logistic regression; SL.gam is a generalized additive model with smoothing splines for the neutralization titer variables; SL.nnet is a neutral network;

SL.polymars is multivariate adaptive polynomial spline regression.

^c^ All = include all variables;

Lasso = include variables with non-zero coefficients in the standard implementation of SL.glmnet optimizing the lasso tuning parameter via cross-validation

Low-collinearity = disallow pairs of quantitative variables (MN, PRNT) with *R^2^* > 0.85;

Univariate log. regression p-value < 0.10 = Wald test p-value in a logistic regression model < 0.10
