# Supplementary material for: Microneutralization assay titer correlates analysis in two phase 3 trials of the CYD-TDV tetravalent dengue vaccine in Asia and Latin America
Source: PLoS One. 2020 Jun 15;15(6):e0234236. doi: 10.1371/journal.pone.0234236 (PMC7295445; doi:10.1371/journal.pone.0234236)
Supplement: S5 Table — (DOCX) [file pone.0234236.s005.docx]

S5 Table. Model terms for the best interpretable model for the Vaccine group, for different data sets. A) Model terms for the best interpretable model for Data Set 4 (demographic + Month 13 PRNT_50_ titer + Month 13 MN titer), a stepwise-selected logistic regression model fit on the full data set. B) Model terms with p < 0.01 for the best interpretable model for the Vaccine group for Data Set 2 (demographic + Month 13 MN titer), a logistic regression model with 2-way interaction terms fit on the full data set.

|  | Coefficient  Estimate | Odds Ratio^a^ | P-value |
| --- | --- | --- | --- |
| A. Model terms for Data Set 4 | | | |
| (Intercept) | -1.50 | 0.22 | 6.20E-09 |
| AGE.12.16^b^ | -0.35 | 0.70 | 1.30E-02 |
| M13.PRNT.S1^c^ | -0.63 | 0.53 | 4.80E-08 |
| M13.PRNT.S2 | -0.79 | 0.45 | 2.70E-09 |
| Sero2.rate^d^ | 1.60 | 4.90 | 3.40E-05 |
| Sero3.rate | 1.00 | 2.70 | 1.70E-03 |
| B. Model terms for Data Set 2 | | | |
| M13.MN.S3:M13.MN.Ave^e^ | -10.00 | 0.00 | 3.90E-05 |
| (Intercept) | -9.40 | 0.00 | 7.70E-06 |
| M13.MN.S3:Sero3.rate | -4.80 | 0.01 | 5.30E-03 |
| AGE.12.16:M13.MN.S3 | -1.70 | 0.18 | 4.20E-03 |
| MALE:M13.MN.S2 | -0.97 | 0.38 | 9.00E-03 |
| MALE:M13.MN.S1 | 1.20 | 3.30 | 4.40E-03 |
| M13.MN.S1:M13.MN.Ave | 4.00 | 52.00 | 4.20E-03 |
| M13.MN.S1:Sero3.rate | 4.40 | 79.00 | 2.60E-04 |
| M13.MN.S2:M13.MN.S3 | 5.00 | 150.00 | 2.60E-05 |
| M13.MN.S3:M13.MN.S4 | 6.10 | 470.00 | 7.90E-05 |
| M13.MN.S3 | 6.70 | 830.00 | 2.80E-04 |

^a^Estimates for interaction terms (indicated by notation X:Y) in the Odds Ratio column are ratios of odds ratios.

^b^Age.12.16 is the indicator of 12-16 years old compared to the reference category 2-5 years old.

^c^M13.PRNT.S1 is Month 13 PRNT_50_ DENV-1 titer, with similar notation for each neutralization assay and serotype.

^d^Sero2.rate is the fraction of placebo group VCD endpoints that are of serotype 2, with similar notation for the other serotypes.

^e^M13.MN.Ave is Month 13 MN average titer to the 4 serotypes.
