# Supplementary figures and images for: Microneutralization assay titer correlates analysis in two phase 3 trials of the CYD-TDV tetravalent dengue vaccine in Asia and Latin America
Source: PLoS One. 2020 Jun 15;15(6):e0234236. doi: 10.1371/journal.pone.0234236 (PMC7295445; doi:10.1371/journal.pone.0234236)

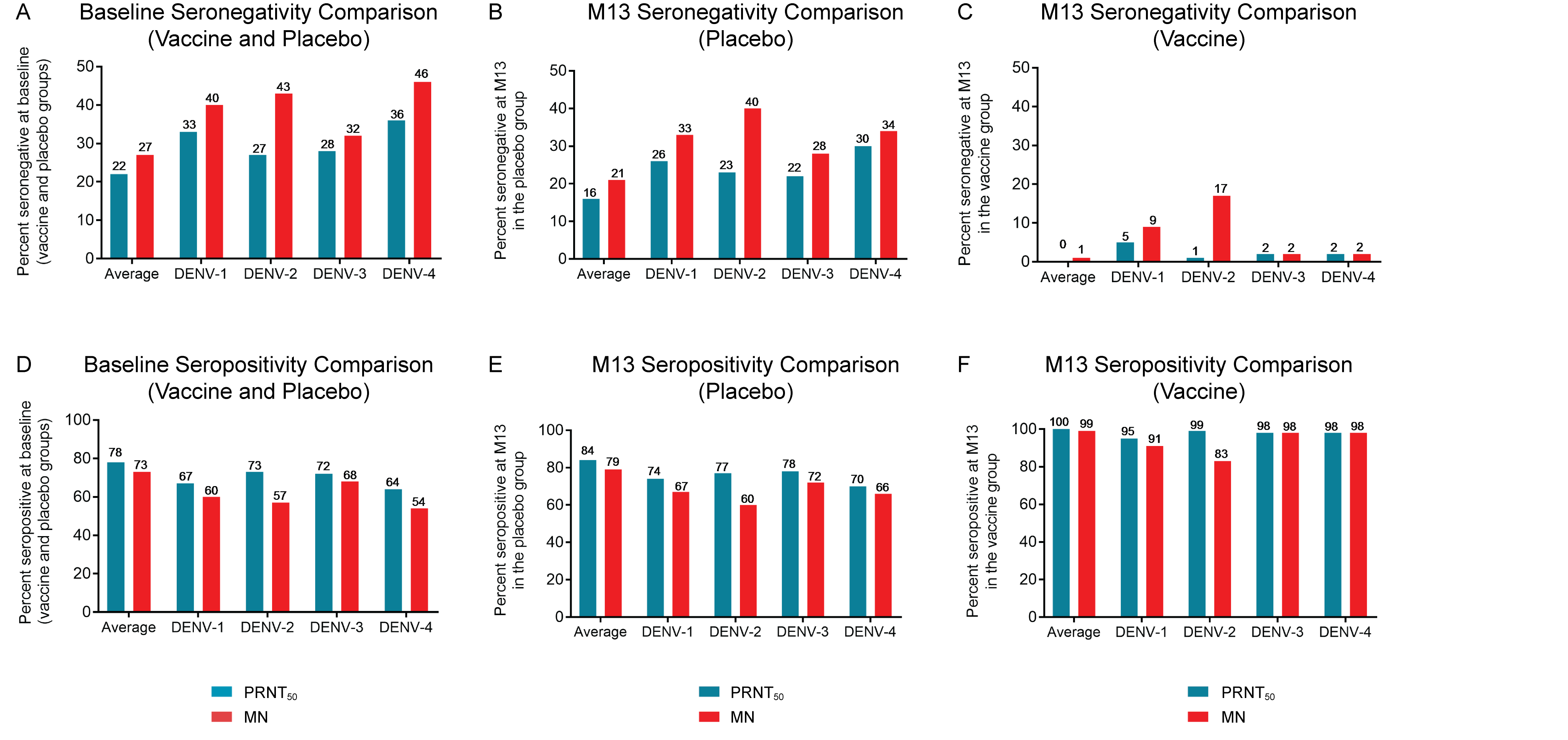

Supplement: S1 Fig — Comparison of classification of CYD14 and CYD15 9–16-year-old immunogenicity subset (A, B) cases and controls or (B, C, E, F) controls as (A, B, C) dengue seronegative vs. (D, E, F) dengue seropositive at (A, D) baseline and at (B, C, E, F) Month 13 according to the PRNT50 (blue) or MN (red) assay. Seropositivity was defined as a titer ≥ 10 for each individual serotype and as a titer ≥ 10 of at least one serotype for the Average readout. Seronegativity was defined as a titer < 10 for each individual serotype and as a titer < 10 for all individual serotypes for the Average readout. (TIF) [file pone.0234236.s006.tif]

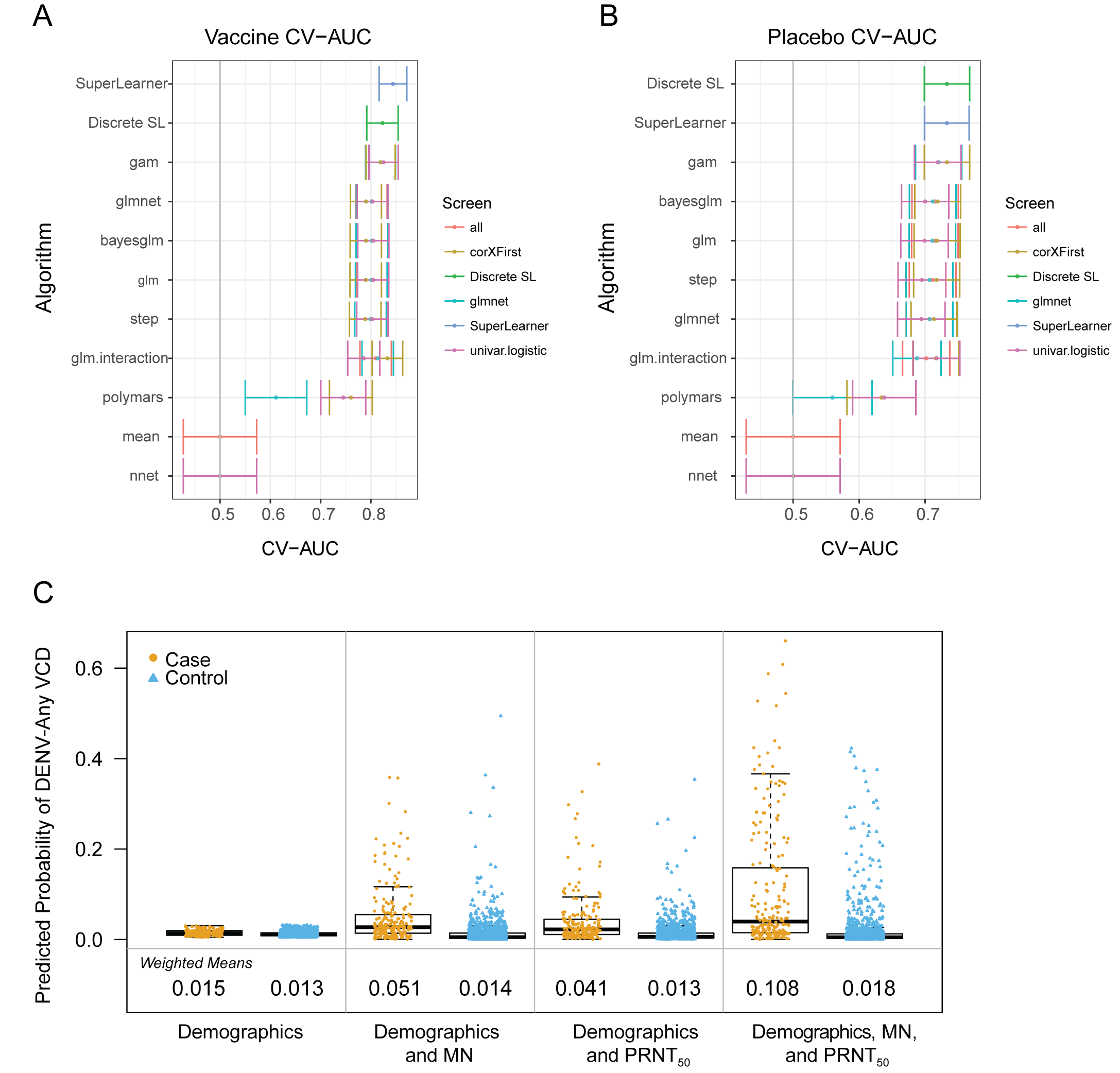

Supplement: S2 Fig — Classification accuracy (A,B) of different algorithms using demographic + MN + PRNT50 data and cross-validated estimated probabilities of DENV-Any by case-control status (C). (A, B): CV-AUC values for classification accuracy of different algorithms using demographic + MN + PRNT50 data as to whether each participant experienced DENV-Any VCD between Months 13 and 25 are shown for (A) the vaccine group and (B) the placebo group for the combined CYD14 and CYD15 9-16-year-old cohort. (C) Cross-validated estimated probabilities of DENV-Any in the vaccine group by case-control status for the best-performing models for each covariate group for the combined CYD14 and CYD15 9-16-year-old cohort. (TIF) [file pone.0234236.s007.tif]
