# Supplementary material for: Microneutralization assay titer correlates analysis in two phase 3 trials of the CYD-TDV tetravalent dengue vaccine in Asia and Latin America
Source: PLoS One. 2020 Jun 15;15(6):e0234236. doi: 10.1371/journal.pone.0234236 (PMC7295445; doi:10.1371/journal.pone.0234236)
Supplement: S1 Text — (DOCX) [file pone.0234236.s008.docx]

S1 Text. Case-cohort sampling design for measurement of Month 13 MN titers in CYD14 and CYD15 participants.

Month 13 MN titers were measured for all Month 13 cases with Month 13 PRNT_50_ titers measured. This meant that Month 13 MN titers were measured in nearly all cases (96.7% in CYD14 and 99.8% in CYD15).

Next, we considered Month 13 immunogenicity subset controls that had Month 13 PRNT_50_ titers measured. For the subgroups of these participants separately in the CYD14 and CYD15 trials, without replacement simple random sampling was done to generate 4 times as many controls as the number of cases sampled within each trial. However, as in the placebo group there was only a 2.6:1 control:case ratio available for sampling in CYD15, a 4:1 sampling for CYD15 would have led to greater than a 4:1 sampling in the vaccine group. Thus, to save resources, for CYD15 we used the control:case ratio expected to yield a 4:1 ratio in the vaccine group, i.e. a ratio of 3.3:1.

For controls with Month 13 PRNT_50_ titer measured, without replacement sampling was done to measure Month 13 MN titer in a 4:1 control:case ratio of participants within each trial.
